# *AP-IO*: Asynchronous Pipeline I/O for Hiding Periodic Output Cost in CFD Simulation

**DOI:** 10.1155/2014/273807

**Published:** 2014-04-03

**Authors:** Ren Xiaoguang, Xu Xinhai

**Affiliations:** State Key Laboratory of High Performance Computing, National University of Defense Technology, Changsha, Hunan 410073, China

## Abstract

Computational fluid dynamics (CFD) simulation often needs to periodically output intermediate results to files in the form of snapshots for visualization or restart, which seriously impacts the performance. In this paper, we present asynchronous pipeline I/O (*AP-IO*) optimization scheme for the periodically snapshot output on the basis of asynchronous I/O and CFD application characteristics. In *AP-IO*, dedicated background I/O processes or threads are in charge of handling the file write in pipeline mode, therefore the write overhead can be hidden with more calculation than classic asynchronous I/O. We design the framework of *AP-IO* and implement it in OpenFOAM, providing CFD users with a user-friendly interface. Experimental results on the *Tianhe-2* supercomputer demonstrate that *AP-IO* can achieve a good optimization effect for the periodical snapshot output in CFD application, and the effect is especially better for massively parallel CFD simulations, which can reduce the total execution time up to about 40%.

## 1. Introduction

Currently, computational fluid dynamics (CFD) is increasingly applied in science and engineering with the continuous development of computer technology, especially the ever-growing computing power of supercomputers [1–3]. In order to meet the needs of visualization or restart, CFD simulation has to periodically output intermediate results to files in the form of* snapshot*.

Unfortunately, this kind of periodic snapshot output has gradually become a performance bottleneck in CFD simulations on massively parallel system. In addition, according to some experimental data [4–6], the periodic large volume snapshot file output can take more than 80% time of the whole CFD simulation process in extreme case. On the one hand, the growth rate of I/O speed is far behind the growth rate of computing capacity [7, 8], which makes I/O become a bottleneck; on the other hand, the requirement of all-refined numerical simulation promotes the mesh scale of many CFD applications to million scale, resulting in the snapshot file tremendous, that is, Gigabytes or even Terabytes [9, 10]. Therefore, it is urgent to optimize the periodic large volume snapshot file output in massively parallel CFD simulations.

Asynchronous I/O is a widely used I/O optimization technique, in which dedicated I/O processes/threads are used to handle the file write operations, and the I/O overhead is hidden by overlapping I/O operations with calculation operations [11, 12]. The key of the asynchronous I/O optimization is to get enough calculation operations to hide the I/O overhead on the premise of data consistency; that is, for any set of data, its asynchronous write operation should be finished before the next update operation.

A CFD simulation, as one kind of typical compute-intensive application, has an immense potential to use asynchronous I/O technology to optimize large-volume file output. However, the snapshot output in traditional CFD simulation always happens at the end of a timestep, which is close to the update operation of the same data at the beginning of next timestep. Thus, there is no enough time for asynchronous I/O to hide the file write overhead.

By analyzing the simulation procedure of CFD applications, which is based on the time step iterations, we find that each timestep's snapshot generally consists of multiple physical data arrays called* field*, and these fields are updated at different time. This potentially allows to output these fields asynchronously in pipeline mode. In this paper, we propose* AP-IO*, a new asynchronous pipeline I/O optimization method for periodic large-volume snapshot output in massively parallel CFD applications. Instead of outputting snapshot's multiple fields together at the end of the time step, AP-IO asynchronously outputs these different fields in a pipeline mode.

The main contributions of this paper are summarized below.

- We introduce* AP-IO*, an asynchronous pipeline I/O method, to solve the I/O performance bottleneck problem of periodic large volume snapshot output in CFD applications.
- We propose an application level calculation segments scheduling method, which schedules the order of the calculation segments of different fields, to obtain more hidden time for* AP-IO*.
- We implement the user-level* AP-IO* framework in the open source CFD software OpenFOAM with the Pthread and compiler-directed instruction techniques.
- We show by experiments on* Tianhe-2* supercomputer with three typical CFD application cases that* AP-IO* optimization method can significantly enhance the performance of periodic large-volume snapshot output in CFD applications. Specifically, the file write overhead can be reduced 60% on average, and the total execution time can be reduced 18% on average.

The rest of the paper is organized as follows.  Section 2 introduces the motivation of our work.  Section 3 introduces the basic idea of* AP-IO*.  Section 4 describes the framework of* AP-IO*, including the programming model, the compiler-directed foundation framework, and the calculation segments scheduling optimization.  Section 5 introduces the implementation of* AP-IO* in OpenFOAM software.  Section 6 describes our evaluation methodology and demonstrates the superiority of* AP-IO* by 3 CFD applications. Finally, Section 7 concludes the paper.

## 2. Motivation

### 2.1. *Snapshot* Output in CFD

**Figure 1:**
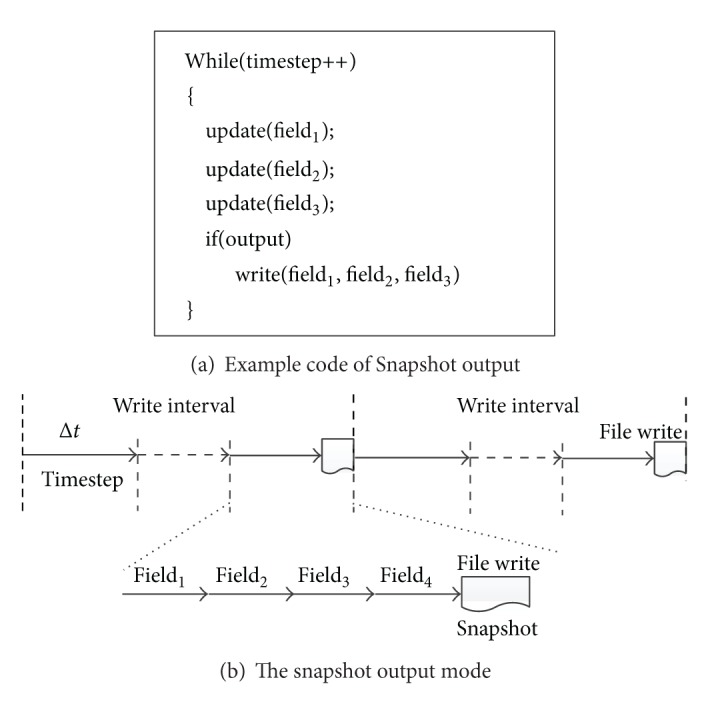
Example code and snapshot output mode of a typical CFD application.

In a typical CFD application, on the one hand, the simulation procedure is discretized into several timesteps, and each timestep is denoted with Δ*t*. It is notable that different timesteps are simulated in the same computing mode, but with different initial condition value. On the other hand, the simulation region is discretized into a mesh system, and the physical property in the simulation region is represented by a* filed*, that is, a data array with the same size of the mesh. As shown in Figure 1(a), each timestep is usually simulated by computing multiple fields one by one. The set of these fields, namely,* snapshot*, is the intermediate simulation result of the timestep.

For the purpose of visualization or restart, a CFD simulation procedure needs to periodically output the snapshot to files on the disk. As shown in Figure 1(b), the snapshot is outputted at the end of* write interval*, which is consisted of fixed number of timesteps.

Nowadays, the mesh size of massively parallel CFD applications reaches million level, and the volume of a snapshot often scales to Gigabyte level. Unfortunately, outputting such large snapshot on HPC system seriously affects the performance of the whole CFD simulation. The reasons are as follows: (a) multiple users or multiple processes competing for shared I/O channel; (b) with the scale of HPC increasing, the performance of computing capability improves more quickly than that of I/O; (c) the redundant data produced by the parallel task partitioning makes the volume of snapshot rise. All these influences will be discussed in detail in Section 6.

According to our experiments as well as in related documents, the file write overhead consumes 20% of the whole CFD simulation time on average, which can reach even more than 80% in extreme circumstances [4–6]. Thus, it is critically important to optimize the periodical large volume snapshot output in massively parallel CFD applications.

### 2.2. Traditional Optimization for File Write

I/O performance optimizations mainly performed in two ways: (a) reducing I/O overhead and (b) hiding I/O overhead.

Buffer mechanism [8], which reduces I/O overhead through buffer caching, is the most universal technology used for reducing I/O overhead in the modern computer system. However, the buffer mechanism is restrained by buffer capacity and difficult to be applied in large-volume file output. In most modern computer systems, the buffer size is less than 1 Megabyte, then the buffer mechanism cannot optimize the CFD snapshot output, which is usually more than hundreds of Megabytes.

**Figure 2:**
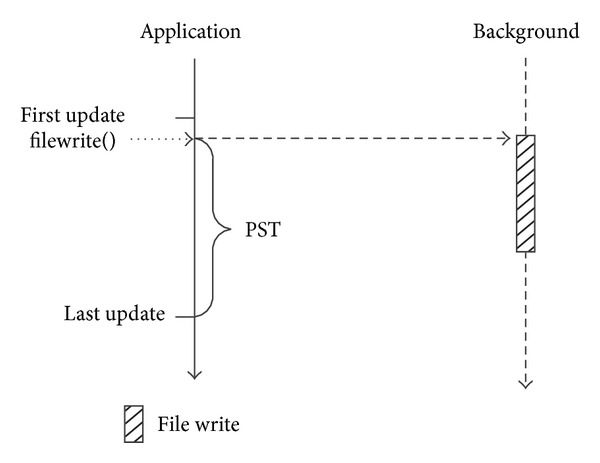
Asynchronous I/O for large volume file.

Asynchronous I/O is another optimization technology used for hiding I/O overhead. The core idea of asynchronous I/O is to use dedicated routines (processes/threads) in background to perform I/O operation, which will hide the file write overhead with calculation operations [13]. As shown in Figure 2, the asynchronous write operations in the background overlap with calculation operations and the file write overhead is hidden. To guarantee the data consistency, for any set of data, the background asynchronous write operation must be finished before the next update operation of the same data.

So, for asynchronous I/O, it is critical that there are enough calculation operations to hide the file write overhead. As a matter of convenience, we define* potential shield time (PST)* as the calculation time used to hide the write overhead. To a data set, if the output operation is closed to the next update operation of the same data, the* PST* will not be sufficient to hide the file write overhead.

## 3. Basic Idea

### 3.1. The Basic Idea of* AP-IO*

**Figure 3:**
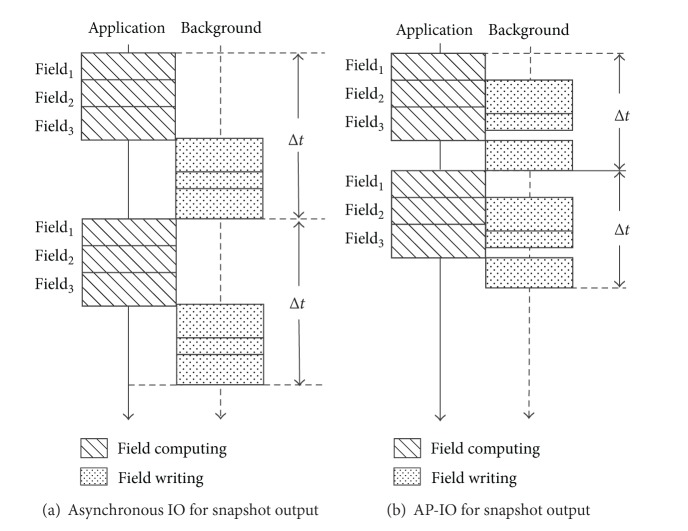
Asynchronous IO versus AP-IO for CFD snapshot output.

In the parallel CFD simulation, there are multiple simulation processes which we call as* compute processes*. As shown in Figure 3(a), it is an example of the asynchronous I/O method applied for snapshot output in the CFD simulation. For each compute process, there is a corresponding dedicated write* service routine* (process/thread) to deal with the file write requests. When a compute process needs to do a write operation, it sends a relevant write request to its corresponding write service routine, which handles the request in background.

A prominent problem of the above traditional asynchronous I/O for the snapshot output in the CFD simulation is the shortage of* PST*. The outputs of all the fields of a snapshot are concentrated at the end of the timestep, which is close to their update operations in the next timestep. In asynchronous I/O, to guarantee the data consistency, these fields' update operations in the next timestep cannot be performed until the asynchronous write operations in the current timestep are completed. This situation makes it clear that there is no* PST* for the asynchronous snapshot output.

**Figure 4:**
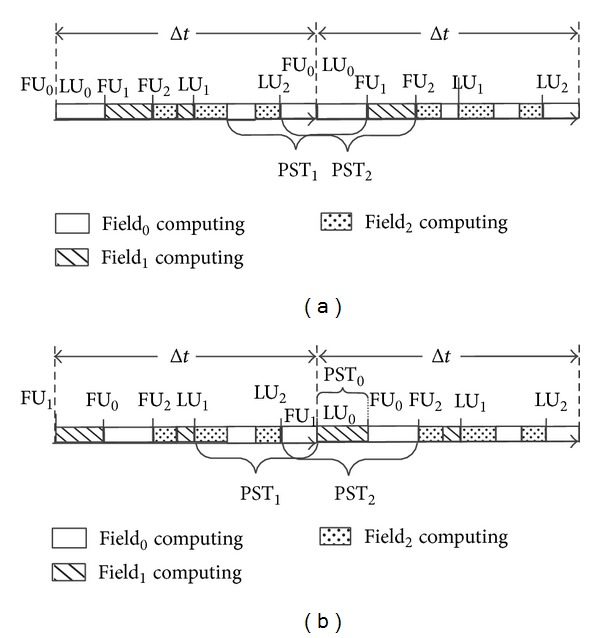
Example of* PST*.

In order to solve this problem, we proposed the* AP-IO* method.* AP-IO* method mainly originates from the updating characteristic of the fields of the snapshot. In general, as shown in Figure 4(a), a field may be updated for several times in a timestep, We call an updating procedure as* calculation segment*, and the calculation segments of a field are stagger with other fields' calculation segments. For a field in a single timestep, we define the* First Update (FU)* time and the* Last Update (LU)* time. The* FU* time of a field is the starting point of the field's first calculation segment in a timestep, and the* LU* time is the end point of the field's last calculation segment in a timestep.

In* AP-IO*, as shown in Figure 3(b), the fields of the snapshot are not outputted together at the end of the time step. On the contrary, for each field, its output follows its* LU* time in the current timestep. To guarantee the data consistency, the field's output operation should be finished before its* FU* time in the next time step. So, the* PST* of a field is the time between the* LU* time in the current timestep and the* FU* time in the next timestep. Compared with the centralized output at the end of timestep,* AP-IO* gets more* PST*, and the field's output operation will be overlap with other fields' calculation operations.

### 3.2. Analysis of* PST*

Providing more* PST* is the key advantage of* AP-IO*, and we will analyze the characteristics of PST in CFD simulation in this section.

The distribution of a field's calculation segments in a single timestep has a direct influence on the length of its* PST* in the* AP-IO* mode. Considering the CFD simulation process shown in Figure 4(a), the *field*_0_'s calculation operations go across the entire timestep. *field*_0_'s* LU* time in the current timestep is overlapped with its* FU* time in the next timestep, resulting in the fact that there is no* PST* for *field*_0_. To obtain more* PST* for *field*_0_, as shown in Figure 4(b), we exchange *field*_1_'s first calculation segment with *field*_0_'s first calculation segment. Now, *field*_0_ can get a* PST PST*_0_ as shown in Figure 4(b).

In order to increase a fields'* PST*, we need to improve the distribution of fields' calculation segments in the snapshot. A calculation segments scheduling optimization method, aiming at improving the distribution of fields, is proposed in this paper, which will be described in Section 4.3.

## 4. Framework

We design the user-level framework of* AP-IO* by combing the compiler-directed technology with the* AP-IO* library, which is convenient for CFD users to carry out the* AP-IO* optimization in their CFD applications.

### 4.1. Programming Model

We define two compiler directives for the* AP-IO* optimization, and their semantics are listed as follows.

- “#FU *field*_1_”: to explicitly identify *field*_1_'s* FU* time, which means when the execution goes across this point, the asynchronous write of *field*_1_ is no longer safe, and it is required to take a blocking wait to guarantee the finish of *field*_1_'s asynchronous write.
- “#LU *field*_1_”: to explicitly identify *field*_1_'s* LU* time, which means that from this point on, the request of *field*_1_'s write can be sent to the background write service routine to perform asynchronous write.

**Figure 5:**
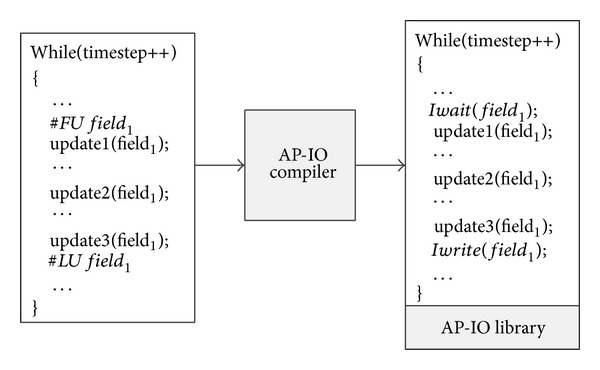
Framework of* AP-IO*.

We will illustrate the* AP-IO* programming model with an example. The left part of Figure 5 shows a typical CFD application code based on timestep iteration. The update procedure of *field*_1_ in the code has three calculation segments: *update*1(), *update*2(), *update*3(). If we use* AP-IO* to optimize the periodic snapshot output, we should add the compile directives in the corresponding position of the code for each field. Take *field*_1_ as an example, we need to add compile directives “#FU *field*_1_” in front of *update*1() and “#LU *field*_1_” after *update*3(), respectively. These two compile directives explicitly define the* PST* of *field*_1_.

This compiler-directive based programming model makes minimum modification of the original CFD application code and enables the CFD application developers to optimize with the* AP-IO* method at minimum cost. It is notable that the compiler directives can be automatically inserted through compile techniques. However, it is not the focus of this paper.

### 4.2. Framework of* AP-IO*

Figure 5 presents our* AP-IO* framework, the core components which include both the* AP-IO* compiler and* AP-IO* library. The* AP-IO* compiler is a compile directives based source-to-source compiler. The CFD application code in the left part of Figure 5 is compiled into the code including* AP-IO* library calls which is shown in the right part of Figure 5.

The* AP-IO* library provides semantic interfaces support for the application code generated by the* AP-IO* compiler. As shown in Figure 5, after preprocessing by the* AP-IO* compiler, the compiler directives, “#LU *field*_1_” and “#FU *field*_1_”, are, respectively, replaced with* AP-IO* library calls: *Iwrite*(*field*_1_) and *Iwait*(*field*_1_), whose semantic functions are, respectively, listed as follows.

- *Iwrite(* *field*_1_): responsible for sending the *field*_1_'s write request to the background service routine. The function will return as soon as the request is successfully sent.* Iwrite()* semantics encapsulate three work: the send of asynchronous write request, FIFO-mode queuing of write requests in the write service routine, background write, and other operations.
- *Iwait(* *field*_1_): responsible for checking whether the corresponding field's asynchronous write operation has been finished. If it has been already finished, a success code will be returned. Otherwise, a blocking wait must be hold until the finish of the asynchronous write operation, to avoid updating the field whose write operation is underway.

### 4.3. Calculation Segments Scheduling Optimization

In order to gain the benefit of* AP-IO*, our AP-IO compiler can also help the developer to optimize the distribution of the calculation segments of each field in a single timestep. Achieving the most optimal scheduling solution is a NP-hard problem. Therefore, we design a heuristic algorithm for the scheduling of field's calculation segments in a single timestep, which is shown in Algorithm 1.

The core idea of our heuristic algorithm is to improve the distribution of each field's calculation segments, that is, reduce the intersection between different fields' calculation segments. First, the scheduling algorithm numbers the fields in a single timestep according to the order of each field's* FU* time. During the scheduling, the algorithm moves the odd number field's segments as close as possible to its first calculation segment, and the even number field's segments as close as possible to its last calculation segment. “As possible” means maintaining the dependencies between the calculation segments, and moving the calculation segments as much as possible to the corresponding direction.

## 5. Implementation of* AP-IO*

We implement the* AP-IO* method in the open source CFD software OpenFOAM with the source-to-source compiler technology and the Pthread technology, which mainly includes two parts: the compiler support layer and the* AP-IO* library.

### 5.1. Compiler Support Layer

With the source-to-source compile technology, the compiler support layer mainly includes two parts: transforming the compiler directives to* AP-IO* library function calls and scheduling the fields' calculation segments in the origin OpenFOAM application code with the heuristic scheduling algorithm in Algorithm 1.

### 5.2. *AP-IO* Library

**Figure 6:**
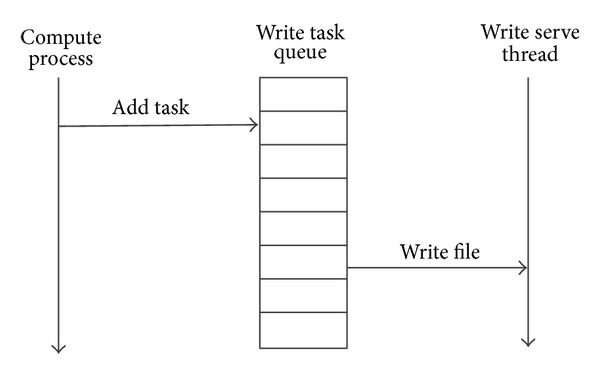
Implement of* AP-IO*.

The* AP-IO* library mainly involves three parts: compute processes, write service threads, and write task queue, as shown in Figure 6.

#### 5.2.1. Iwrite

First, a Pthread thread is created for each parallel simulation process (compute process) as the write service thread. A write task queue is shared by each compute process and its write service thread. When the compute process trigger the function call* Iwrite()*, a write request will be added to the write task queue. The write service thread in the background constantly scans the write task queue, and all the tasks in the queue are processed in FIFO mode.

A status variable *write*_*state* is set for each field, and it has three states: *WAIT*_*WRITE*, *WRITING*, and *WRITE*_*FINISHED*, the meanings of them are listed as follows.

- *WAIT*_*WRITE* means that this field's write request in the write task queue is waiting to be handled by the write service thread.
- *WRITING* means that this field's write request is being handled by the write service thread and this field is writing in the background now.
- *WRITE*_*FINISHED* means that this field's write request had been finished, or there is no write request for this field.

For a field, the default status of* write_state* is *WRITE*_*FINISHED*. When the function* Iwrite(* *field*_1_) is called in compute process, the write request of *field*_1_ is added into the write task queue, and* write_state* of *field*_1_ is set as *WAIT*_*WRITE*. When the write request begins to be handled by the write service thread,* write_state* of *field*_1_ will be set as *WRITING*. Finally, the status will be set to *WRITE*_*FINISHED* when the write request is finished.

#### 5.2.2. Iwait

To guarantee the data consistency, the compile-directive “#FU *field*_1_” explicitly specifies the opportunity of checking whether *field*_1_'s write has been finished. The function call* Iwait()* in the compute process is in charge of the checking work. The checking work of* Iwait()* is performed by reading the status of *write*_*state*. If the status of *write*_*state* is *WRITE*_*FINISHED*,* Iwait()* returns with a success code. Otherwise, a blocking wait is launched and the waiting procedure will not finish until the status of *write*_*state* is updated to *WRITE*_*FINISHED*.

#### 5.2.3. Threading Optimizations

The above mechanism can basically perform the* AP-IO* optimization in the OpenFOAM platform, but there are two situations that should be taken into account for the CPU consumption, which influence the performance optimization.

**Figure 7:**
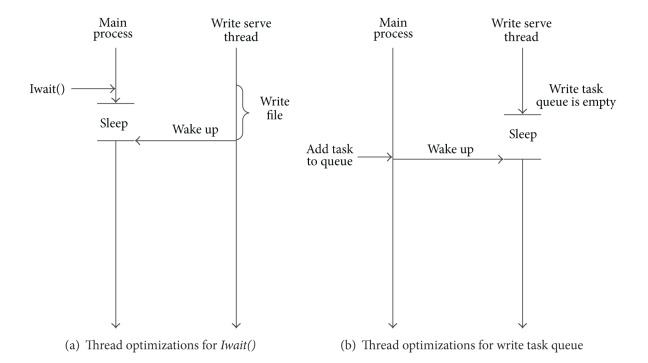
Threading optimizations for* AP-IO*.

The first situation is shown in Figure 7(a). In the* Iwait()* function call, if the status of* write_state* is *WRITING* or *WAIT*_*WRITE*, a blocking wait is needed. This blocking wait has two implementation ways. One is to constantly check the status of* write_state* with* while* iteration, which will seriously consume CPU resources, resulting in low efficiency. So, we take another more efficient way. In the procedure of the blocking wait, the compute process is suspended and sleeping to release CPU resources. Once the write service thread finishes the current write request, it awakens the sleeping compute process by sending a signal to it. Therefore, the compute process can greatly reduce the CPU resources consumption during the sleep time.

The second situation is shown in Figure 7(b), it is related to the write service thread's scan of the write task queue. When the queue is empty and no write request needs to be handled, to timely responds to the new write request, the write service thread has to pay close attention to the change of the write task queue. Similar to the first situation, once the write service thread finds that the write task queue is empty, it suspends itself to sleep and releases CPU resources. The compute process always tries to send an awake signal to the write service thread when a new write request arrives. If the write service thread is sleeping, it will be awakened at the time of new request's arrival.

The two suspend/awake mechanism can ensure that the compute process and the write service thread will not consume too many CPU resources, which guarantees the efficient execution of* AP-IO* in CFD simulation.

## 6. Experiment

We demonstrate the superiority of our* AP-IO* method on the Tianhe-2 supercomputer with three typical CFD application cases.  Section 6.1 introduces the computer platform Tianhe-2 used in the experiments.  Section 6.2 discusses the benchmarks selected.  Section 6.3 describes the experiment environment and the evaluation methodology.  Section 6.4 presents and analyzes our results.

### 6.1. Platform

The* Tianhe-2* supercomputer was built by China's National University of Defense Technology (NUDT), which is the world's fastest supercomputer with a 33.86 petaflop peak performance according to the TOP500 list in June 2013 [14]. Our experiments use a subsystem of* Tianhe-2* with 128 compute nodes as the test platform, and each computing node comprises two Intel Ivy Bridge Xeon processors, three Xeon Phi chips (accelerator card), and 64 Gigabyte DRAM [15]. It should be pointed out that our experiments only use the processors, and do not use the accelerator card.

The whole I/O subsystem of* Tianhe-2* has a disk array with a 12.4 Petabyte capacity, and all the nodes in the system share the disk array through a global interconnecting network. The interconnecting network uses the optoelectronics hybrid transport technology, connecting all the compute nodes through 576 connection ports with 13 large routers, and the transmission rate reaches 6.36 GB/s.

It is noticeable that although the subsystem we use is exclusive for us, the I/O system is globally shared with other users, which can lead to competition with other users and impact the precision of the experimental results. A detailed analysis of this problem will be described in Section 6.3.1.

### 6.2. Benchmark

To verify the superiority of* AP-IO*, we select three typical large-scale CFD applications as benchmarks. They are* dambreak*,* cavity,* and* pitzDaily*. The three benchmarks are briefly introduced as follows.

- *Dambreak*:* dambreak* is a simulation of dambreak based on the volume of fluid (VOF) method. The two-phase flow solver* interFoam* in OpenFOAM is used.
- *Cavity*:* cavity* is the lid-driven cavity flow simulation, using the incompressible flow solver* icoFoam* in OpenFOAM.
- *PitzDaily*:* pitzDaily* is a large eddy simulation (LES) case. The solver used is* pisoFoam* together with the* oneEqEddy* eddy viscosity model.

Some important parameters of the three benchmarks are shown in Table 1. All the benchmarks are in 3D with the grid scale between 5 M and 17 M. To verify the effect of the write interval to the performance of snapshot output, we tested five write interval conditions, including the none condition with no snapshot output. All the three benchmarks take* Scotch* as the decompose method for parallel grid partition.

### 6.3. Experiment Design

#### 6.3.1. Experiment Environment Analysis

**Figure 8:**
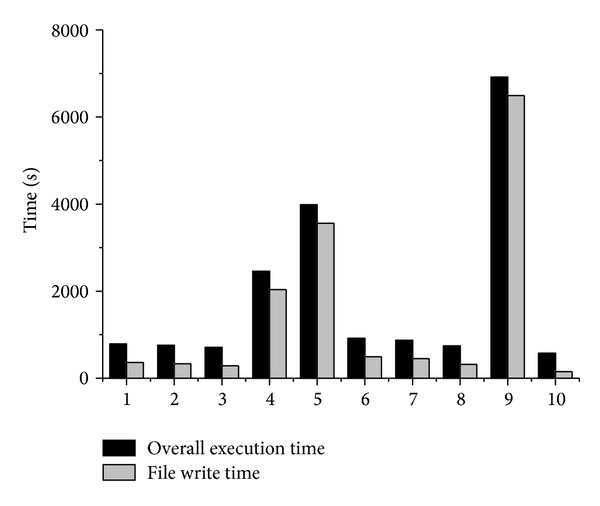
The execution time of* cavity* runs on* Tianhe-2* for 10 times.

As mentioned in Section 6.1 that we use the I/O subsystem shared with other users, our experimental results are more or less random. To measure this randomness, we test the* cavity* benchmark for 10 times with 256 processors. The test is performed in a continuous time, during which the* Tianhe-2* system is running hundreds of other users' tasks at the same time, and these tasks may also perform file write operations to the disk array. The execution times are shown in Figure 8.

According to Figure 8, we can find that the execution times of the 4th, 5th, and 9th test are much higher than those of others. The gap reaches 10 times, which indicates severe abnormalities. Therefore, to eliminate the influence on I/O performance from the multiuser and multitask environment, we perform a test for multiple times and choose the average of the execution time as experiment result. Generally speaking, all the following experiments results are the average value of multiple tests.

#### 6.3.2. Evaluation Methodology

The purpose of our experiments is to obtain the performance optimization effect of* AP-IO* in OpenFOAM by comparison of results before and after the optimization. The tests are performed in two dimensions: multiple numbers of processors and multiple write intervals. Our tests start from 4 processors and scale to 1024 processors with a scale coefficient of 2. Meanwhile, to verify the write interval's effect on I/O performance, we have to get all the benchmarks tested, respectively, with 1Δ*t*, 2Δ*t*, 4Δ*t*, 10Δ*t*, and none (no snapshot output) write interval conditions.

Our experiments mainly tested three indicators of the performance: the growth of the output file's volume that scales with the number of processors, the original file write overhead without* AP-IO* optimization, and the optimization effect with* AP-IO* method. The first two aspects mainly analyze the I/O characteristics of the tree benchmarks, which will be taken as the baseline. The third aspect is to measure the effect of* AP-IO* optimization directly.

First, to measure the growth of the output volume with the influence of the number of processors, we get the three benchmarks tested under the number of processors ranging from 2 to 1024 and four write interval conditions (1Δ*t*, 2Δ*t*, 4Δ*t*, and 10Δ*t*). Recording the volume of the output file, we will get the characteristics of file volume growth due to the parallel grid partition.

Second, we have to measure the overhead of the periodic snapshot output without* AP-IO* optimization. Suppose that the execution time under the none write interval condition is *T*_0Δ*t*_, and the execution time under *n*Δ*t* write interval condition without optimization is *T*_*n*Δ*t*_^prim^, then the file write overhead *T*_file_^prim^ can be approximated by the following methods as

(1) $$\begin{array}{c} T_{\text{file}}^{\text{prim}}=T_{n\Delta t}^{\text{prim}}-T_{0\Delta t}. \end{array}$$

Finally, the optimization effect of* AP-IO* is measured with two indicators: one is the optimization ratio to the snapshot output overhead, and the other is the optimization ratio to the total simulation time. We define the total execution time of the simulation with* AP-IO* under *n*Δ*t* write interval condition as *T*_*n*Δ*t*_^opt^, and then the file write overhead with* AP-IO* optimization *T*_file_^opt^ can be approximated as s

(2) $$\begin{array}{c} T_{\text{file}}^{\text{opt}}=T_{n\Delta t}^{\text{opt}}-T_{0\Delta t}. \end{array}$$

The optimization ratio of* AP-IO* to the file write overhead *α* is

(3) $$\begin{array}{c} \alpha =\frac{T_{\text{file}}^{\text{prim}}-T_{\text{file}}^{\text{opt}}}{T_{\text{file}}^{\text{prim}}}. \end{array}$$

The optimization ratio of* AP-IO* to the total simulation time *β* is

(4) $$\begin{array}{c} \beta =\frac{T_{n\Delta t}^{\text{prim}}-T_{n\Delta t}^{\text{opt}}}{T_{n\Delta t}^{\text{prim}}}. \end{array}$$

### 6.4. Results and Analysis

#### 6.4.1. Volume of the Snapshot Output

**Figure 9:**
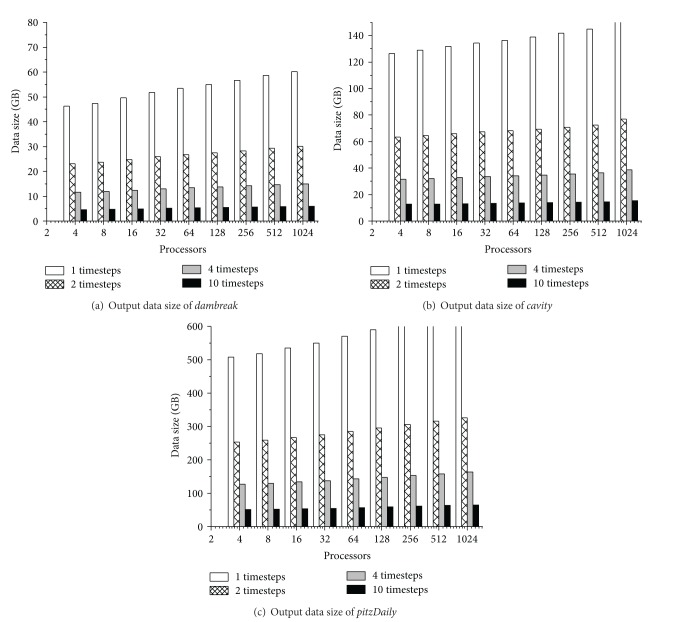
CFD application file output volume increases with scale of the number of processors.

As shown in Figure 9, they are the total volumes of the three benchmarks' (*dambreak, cavity, and pitzDaily*) snapshot output tested with the number of processors from 2 to 1024 under different write interval conditions. We can find that the total volume scales with the same rate as the number of processors grows; the rate is about 2% for* dambreak*, about 6% for* cavity*, and 3% for* pitzDaily*. The growth of the volumes is mainly due to the data redundancy in the grid and fields produced by parallel grid domain decompose. Although the growth rate is less than 10%, it still has severe influence on I/O performance, which will be verified and analyzed in Section 6.4.2.

#### 6.4.2. File Write Overhead

**Figure 10:**
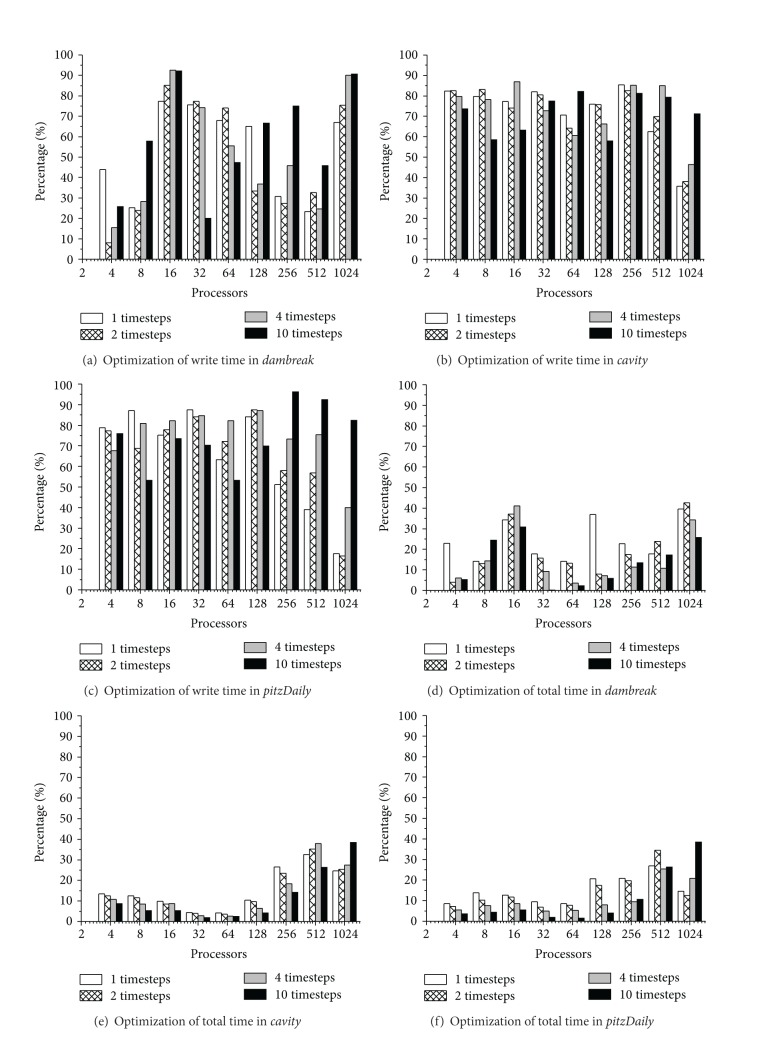
Overhead and proportion of periodical file write.

To get the baseline of the* AP-IO* optimization, we first have to measure the periodical snapshot output overhead for the entire simulation.  Figure 10 shows the three benchmarks' file write overhead.

**Figure 11:**
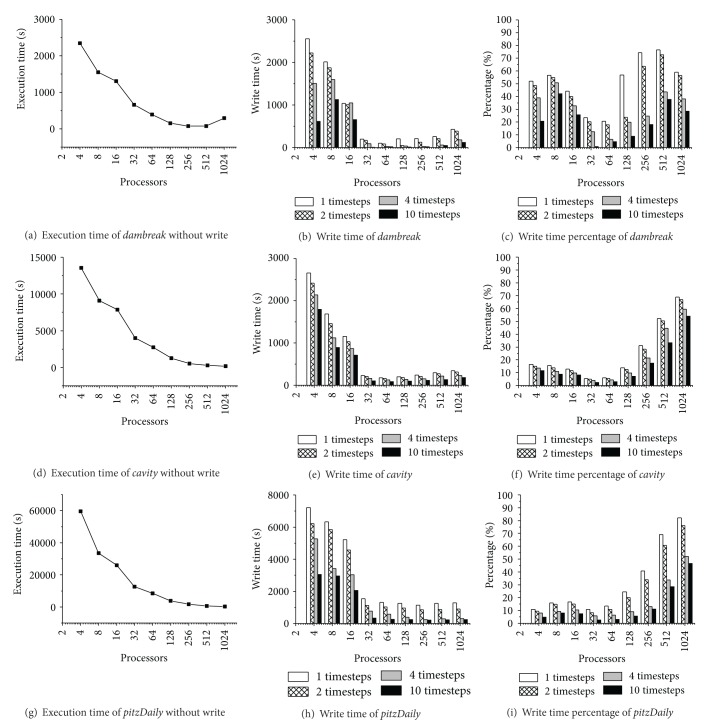
Optimization effect of AP-IO.

As mentioned before, we measure the file write overhead indirectly with the difference between the execution time under certain write interval conditions and the none write interval condition. Figures 11(a), 11(d), and 11(g) are the execution time of the three benchmarks under the none write interval condition, respectively. It can be found that the performance improves as the number of processors increases, but the scalability is limited at 512 processors.

Figures 11(b), 11(e), and 11(h) show the three benchmarks' file write overhead under different write interval conditions and the number of processors. It can be found that the file write overhead declines with the scale of the number of processors firstly. This phenomenon is mainly due to the fact that the write operations are also performed in parallel with the parallel of processors. On the premise that the I/O channel bandwidth is sufficient, the snapshot output speed scales with the number of processors. But when the number of processors scales to a certain value (64 in our results), the I/O channel bandwidth is not sufficient, and the competition caused by the concurrent write operations makes the write speed decline.

Figures 11(c), 11(f), and 11(i) show how the three benchmarks' proportion of file write overhead in the total simulation time scales with the number of processors. It can be found that although the write file overhead declines with the scale of the number of processors, the proportion of the overhead in the total simulation time ascends. This is due to the fact that the growth of the number of processors makes the file write overhead descend, but its descending speed is far less than that of the calculation time, which makes the proportion of the snapshot output overhead in total simulation time show an ascending tendency. It is notable that the snapshot output overhead portion of* dambreak* at 1024 processors is lower than that at 512 processors in Figure 10(c), which is due to the rise of calculation time caused by this benchmark's negative speedup at 1024 processors.

#### 6.4.3. Optimization Effect of* AP-IO*

Figure 11 shows the optimization effect of* AP-IO*, which is measured with the optimizing effect on the snapshot output overhead and the total simulation time, respectively. As shown in Figures 10(a)–10(c), the optimization ratio of snapshot output overhead can reach more than 50% in average and 90% in the best case.

As shown in Figures 11(d)–11(f), the optimization ratio of the total simulation time can reach to 10% with* AP-IO* on average. However, as the snapshot output overhead is hidden by the calculation operation, this optimization ratio of the total simulation time cannot exceed 50%. The trends in the figure show that* AP-IO* is more effective for the simulations under the intensive write interval condition. The optimization effect of the total simulation time tends to decrease when the number of processors is smaller than 64. However, when the number of processors is more than 64, the optimization effect of the total simulation time tends to increase. This phenomenon agrees with the trends of the file write overhead ratio in the total simulation time shown in Figure 10.

The experimental results show that* AP-IO* can well optimize the periodic snapshot output in CFD simulation and gains better optimization effect in massively parallel simulations.

## 7. Conclusions

We have proposed an* AP-IO* optimization method based on the features analysis of CFD simulation for the problem of periodic large volume snapshot output in CFD simulation. We design the* AP-IO* framework for CFD application by combining the compiler-directed technology and the application library. We implement* AP-IO* with multithreading technology in the open source CFD software OpenFOAM. Experimental results demonstrate that our* AP-IO* optimization technique can achieve a good optimization effect for the periodical snapshot output in CFD simulation, which can reduce 10% of the total execution time on average.

## Figures and Tables

**Algorithm 1:**
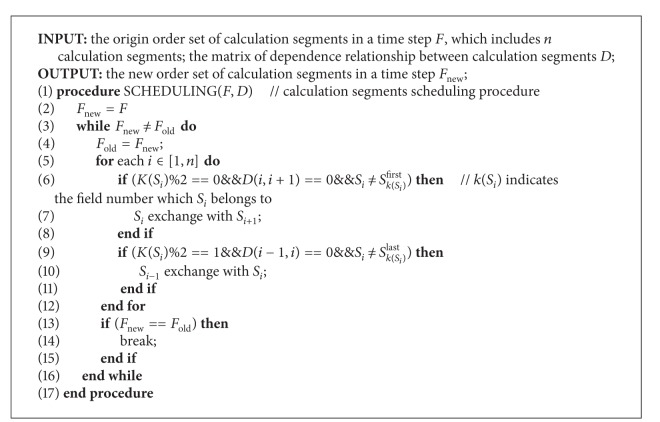
A heuristic algorithm for the scheduling of fields' calculation segments in a single timestep.

**Table 1:** The parameters of the three benchmark.

	Dambreak	Cavity	PitzDaily
Solver	interFoam	icoFoam	pisoFoam
Dimensionality	3D	3D	3D
Number of cells	6229504	15625000	16131636
Δ*t*	1.00*E* − 03	1.00*E* − 04	2.00*E* − 04
Number of time steps	100	100	100
Write interval (time Steps)	1/2/4/10/none	1/2/4/10/none	1/2/4/10/none
Number of fields	5	8	8
Decomposition method	Scotch	Scotch	Scotch

## References

[1] Roose D, Vandriessche R (1995). *Parallel Computers and Parallel Algorithms for CFD: An Introduction*.

[2] Norton T, Sun D-W (2006). Computational fluid dynamics (CFD)—an effective and efficient design and analysis tool for the food industry: a review. *Trends in Food Science and Technology*.

[3] Iserles A (1993). Parallel computational fluid dynamics: implementation and results. *Journal of Fluid Mechanics*.

[4] No J, Thakur R, Choudhary A (2003). High-performance scientific data management system. *Journal of Parallel and Distributed Computing*.

[5] Mickler H, Knüpfer A, Kluge M, Müller MS, Nagel WE (2009). Trace-based analysis and optimization for the semtex CFD application—hidden remote memory accesses and I/O performance. *Euro-Par 2008 Workshops—Parallel Processing*.

[6] Lim GP, Yang F, Chu T, Chen P (2000). Ibuprofen suppresses plaque pathology and inflammation in a mouse model for Alzheimer's disease. *The Journal of Neuroscience*.

[7] Ma X, Winslett M, Lee J, Yu S Improving MPI-IO output performance with active buffering plus threads.

[8] Ma X, Lee J, Winslett M (2006). High-level buffering for hiding periodic output cost in scientific simulations. *IEEE Transactions on Parallel and Distributed Systems*.

[9] Mavriplis DJ, Pirzadeh S (1999). Large-scale parallel unstructured mesh computations for 3D high-lift analysis. *Journal of Aircraft*.

[10] Gropp WD, Kaushik DK, Keyes DE, Smith BF (2001). High-performance parallel implicit CFD. *Parallel Computing*.

[11] Iyer S, Druschel P (2001). Anticipatory scheduling: a disk scheduling framework to overcome deceptive idleness in synchronous I/O. *SIGOPS: Operating Systems Review*.

[12] Acharya A, Uysal M, Bennett R Tuning the performance of I/O-intensive parallel applications.

[13] Ali N, Carns P, Iskra K Scalable I/O forwarding framework for high-performance computing systems.

[14] http://www.top500.org/.

[15] Dongarra J (2013). *Visit to the National University for Defense Technology*.

